# Performance of clinical guidelines compared with molecular tumour screening methods in identifying possible Lynch syndrome among colorectal cancer patients: a Norwegian population-based study

**DOI:** 10.1038/sj.bjc.6605509

**Published:** 2010-01-05

**Authors:** G Tranø, W Sjursen, H H Wasmuth, E Hofsli, L J Vatten

**Affiliations:** 1 Department of Surgery, Levanger Hospital, Sykehuset Innherred, Kirkegata 2, Levanger 7600, Norway; 2 Department of Pathology and Medical Genetics, St. Olavs Hospital, Trondheim University Hospital, Norway; 3 Department of Laboratory Medicine Children's and Women's Health, Norwegian University of Science and Technology, Olav Kyrresgt 17, Trondheim 7006, Norway; 4 Department of Gastrointestinal Surgery, St. Olavs Hospital, Trondheim University Hospital, Olav Kyrresgt 17, Trondheim 7006, Norway; 5 Department of Oncology, St. Olavs Hospital, Trondheim University Hospital, Olav Kyrresgt 17, Trondheim 7006, Norway; 6 Faculty of Medicine, Department of Public Health, Norwegian University of Science and Technology, Olav Kyrresgt 17, Trondheim 7006, Norway

**Keywords:** Lynch syndrome, Revised Bethesda Guidelines, Amsterdam II criteria, microsatellite instability, MMR gene promotor methylation, BRAF oncogene

## Abstract

**Background:**

The aim of this study was to assess the performance of the Revised Bethesda Guidelines (RBG) and the accuracy of the Amsterdam II criteria (AM II) in identifying possible Lynch syndrome (LS) compared with the results of molecular tumour testing.

**Methods:**

Tumours from 336 unselected colorectal cancer patients were analysed by three molecular tests (namely microsatellite instability (MSI), *BRAF* mutation and methylation of mismatch-repair genes), and patients were classified according to the RBG and AM II criteria.

**Results:**

A total of 87 (25.9%) patients fulfilled the RBG for molecular tumour analyses (MSI and/or immunohistochemistry), and the AM II identified 8 (2.4%) patients as having possible LS. Molecular tests identified 12 tumours (3.6%) as probable LS. The RBG identified 6 of the 12 patients (sensitivity 50%), whereas 5 of the 8 patients who fulfilled the AM II criteria were not likely to be LS, based on molecular tests (predictive value of positive test, 38%).

**Interpretation:**

Assuming a fairly high accuracy of molecular testing, the performance of the RBG in identifying patients with possible LS was poor, and the AM II criteria falsely identified a large proportion as having possible LS. This favours the use of molecular testing in the diagnosis of possible LS.

Colorectal cancer (CRC) is the second most common cancer in Norway, with ∼3500 new patients diagnosed annually in a population of 4.7 million people (Cancer Registry of Norway; http://www.kreftregisteret.no). It has been suggested that up to 20–25% of CRC cases are familial, including heredity caused by identified genetic factors and familial clustering of CRC in which the hereditary patterns are complex or unknown (de la Chapelle, 2004). Lynch syndrome (LS) constitutes 3–5% of all CRCs (Lynch *et al*, 2004). In individuals with this autosomal dominant predisposition, CRC tends to develop ∼20 years earlier than sporadic CRC, and the lifetime risk for CRC is 50–80% (de la Chapelle, 2004; Strate and Syngal, 2005). Patients with LS and family members also have an increased risk of other LS-related tumours (Tables 1 and 2) (de la Chapelle, 2004; Lynch *et al*, 2004). In 1993, it was established that the underlying genetic causes of LS were germline mutations in DNA mismatch-repair (MMR) genes (de la Chapelle, 2004; Strate and Syngal, 2005).

The Amsterdam II criteria (AM II) (Table 1) are currently used to identify patients with possible LS, whereas the Revised Bethesda Guidelines 2004 (RBG) (Table 2) were developed to select CRC patients for molecular tumour analyses (microsatellite instability (MSI) and/or immunohistochemistry (ICH). If the results of these molecular analyses indicate LS, it is recommended to carry out germline testing of DNA MMR genes to confirm the diagnosis (Vasen *et al*, 2007; Julie *et al*, 2008).

Approximately 95% of LS-associated tumours display a high level of MSI (MSI-H), but 10–20% of sporadic CRCs are also characterised by MSI (Young *et al*, 2001; Kambara *et al*, 2004), and therefore, isolated MSI-H analyses cannot reliably identify LS-related tumours. However, it has been shown that methylation of MMR genes (such as *MLH1*, *MSH2*, *MSH6* and *PMS2*) is an underlying mechanism of sporadic MSI-H CRC, and methylation status in combination with MSI could therefore be useful in discriminating sporadic from LS-associated CRC (Young *et al*, 2001; Hitchins *et al*, 2007). Exceptionally, microsatellite stable (MSS) tumours with no *BRAF* mutation and no methylation of MMR genes may be LS associated (Julie *et al*, 2008), and caused by the less penetrant *MSH6* gene that is mutated in approximately 5–10% of LS tumours, whereas mutations of *MLH1* and *MSH2* account for approximately 90–95% of LS tumours (de la Chapelle, 2004). Mutation of the *BRAF* oncogene in tumour tissue nearly excludes the possibility of LS (Deng *et al*, 2004; Koinuma *et al*, 2004).

Therefore, we supplemented MSI analyses with the *BRAF* mutation and methylation analyses of MMR genes, and used a combination of these three molecular tests to discriminate colorectal tumours that are likely to be associated with LS from tumours that are not likely to be associated with LS (Deng *et al*, 2004; Domingo *et al*, 2004; Koinuma *et al*, 2004; Vasen *et al*, 2007). The aim of the study was to assess the performance of the RBG in selecting MSI-H CRC tumours that in most cases are associated with LS, and to assess the accuracy of the AM II in identifying patients with possible LS tumours against the results of molecular tests. The study was conducted among unselected CRC patients from two hospitals in Norway (Tranø *et al*, 2009).

## Materials and methods

From January 2007 to June 2008, 336 unselected and consecutively admitted patients with newly diagnosed CRC were recruited from two hospitals in Norway (Tranø *et al*, 2009). The female–male ratio was 0.94, and the mean age at diagnosis was 70 years (range: 29–99 years).

Each patient was interviewed and all relevant data from their medical records were collected. The interviews included a detailed family history of CRC and LS-related tumours or of any other cancer in the family, as described previously (Tranø *et al*, 2009). Pedigrees were traced vertically and laterally as far as possible. If patients reported family members with suspected CRC or ‘abdominal cancer’, or if cancer of internal genital organs was reported in female relatives, the patients and their families were classified as having a negative family history of CRC or other LS-related tumours (Table 2). Only patients who felt confident about their relatives’ medical histories were classified with a positive family history. We did not search in cancer registers or relatives’ medical records for verification of the information obtained from interviews. Data collected from medical records included history of cancer, family history, age, gender and histopathological reports.

From all patients who underwent surgery or those who had a biopsy, 354 malignant tumours in 336 patients were examined for histological features reported to be associated with possible LS (Young *et al*, 2001; Jenkins *et al*, 2007). A total of 14 (4.2%) patients (Table 3) had synchronous cancers; 3 of them had more than two tumours. All pathological examinations were conducted by two experienced pathologists (Tranø *et al*, 2009) and if the pathology reports documented in the medical records differed from the conclusions of the study pathologists, we used the latter conclusions. All elements of the RBG (Table 2) and the AM II (Table 1) were assessed, and each patient was classified according to both sets of clinical criteria.

Molecular tests (MSI, *BRAF* and methylation analyses) were performed on 354 tumours obtained from 336 patients using paraffin-embedded tumour specimens, and control DNA from each patient was obtained from normal colorectal mucosa or from peripheral blood. Patients with synchronous CRC were classified as fulfilling the RBG if one of the tumours indicated possible LS according to molecular tests. Tumours were classified as microsatellite unstable (MSI-H) if two or more of six microsatellite markers showed instability, and as MSS if no more than one of the markers showed instability. No tumours were classified as those with low MSI in this study. The MSI status was based on six recommended microsatellite markers: TGF-*β*-IIR, BAT25, BAT40, D5S107, D5S406 and D13S153 (Boland *et al*, 1998; Hendriks *et al*, 2006). All tumours were also tested for mutation of the BRAF oncogene V600E, and methylation analyses of MMR genes associated with LS were also performed (in the study, only methylation of the *MLH1* gene was found). Using these molecular test results, tumours were either classified as likely to be LS, or not likely to be associated with LS. The molecular analyses and laboratory techniques have been described previously (Aaltonen *et al*, 1998; Boland *et al*, 1998; Loukola *et al*, 2001), and a summary table is provided in Appendix A. Non-LS-associated tumours are likely to be sporadic in the majority of cases, but other genetic factors not related to LS may be present.

To secure blinded interpretation, information obtained from medical records or from interviews on family history, or information about fulfilment of clinical guidelines (RBG, AM II), was not disclosed to the pathologists or to the molecular geneticists who were responsible for histopathological interpretations and molecular tumour analyses. Similarly, molecular tumour test results were not communicated to the clinician before classification of patients according to RBG, except for the histopathological features that are required for assessing patients according to the third criterion of the RBG (Table 2). Using the results of the three molecular tumour tests as the ‘gold standard’, we estimated sensitivity, specificity and positive predictive values (PPVs) for the RBG and the AM II criteria. Statistical analyses were conducted using SPSS for Windows, version 14.0 (SPSS Inc., Chicago, IL, USA).

Written informed consent to carry out molecular tumour analysis was obtained from 336 patients. Nine patients were unable to consent or provide any relevant information because of dementia, incapacity to understand the language, psychiatric disease or critical illness, and hence were not eligible to participate. Two invited patients refused to participate. The study was approved by the Regional Committee for Ethics in medical research and by the Norwegian Data Inspectorate.

## Results

A total of 12 (3.6%) tumours from 12 patients were highly indicative of LS, based on molecular test results. These tumours were MSI-H, and had no BRAF (V600E) mutations or methylation of MMR genes. The remaining 324 (96.4%) tumours had molecular combinations not likely to be indicative of LS. Patients’ demographics, family histories and tumour characteristics from the 12 patients whose tumours were identified as likely to be LS associated according to the molecular methods are listed in Table 4.

Among all patients, 57 (17.0%) had MSI-H tumours and 279 (83.0%) had MSS tumours. There was no *BRAF* mutation in 280 (83.3%) tumours, and 260 (92.9%) of these tumours were MSS. The *BRAF* mutation was detected in 58 (17.3%) tumours: 19 (32.8%) of these were MSS and 39 (67.2%) were MSI-H. In 45 (13.4%) tumours, *MLH1* methylation was found, all of which were MSI-H.

The combination of an MSS tumour without a *BRAF* mutation and no methylation of the *MLH1* gene was observed in 260 tumours, and 19 tumours had a combination of MSS with *BRAF* mutation and no *MLH1* methylation. A total of 45 MSI-H tumours were classified as not being associated with LS because they had *MLH1* methylation; 39 of these tumours also had *BRAF* mutation, whereas 6 had no *BRAF* mutation. The majority of these 45 tumours were found in patients with no family history of CRC, the patients were elderly (mean age: 74.3 years) and the majority were women (62.2%). The tumours tended to be right sided (84.4%), mucinous (55.6%) and with low-grade differentiation (48.9%). The molecular test results are listed in Table 5.

Among the 336 patients, 8 (2.4%) fulfilled the AM II criteria for LS. Although the AM II identified five of the eight patients as having possible LS, the tumours were not LS associated according to molecular tests (PPV 38%). Only three of the eight AM II-positive patients (sensitivity 25%) had molecular test results suggesting LS (Table 6). Clinical characteristics, the results of molecular tumour analyses and family history of patients fulfilling the AM II criteria are listed in Table 7.

On the other hand, among 324 CRC patients who were not classified as having LS by molecular tumour testing, the AM II criteria classified 319 as not being associated with LS (specificity 98%).

A total of 87 (25.9%) patients (44 men and 43 women) were selected by the RBG for further analysis of MSI or IHC with the intention to identify LS. In all, 6 of the 12 patients whose tumours were identified by molecular tumour test results fulfilled the RBG (sensitivity 50%) (Table 6). Among 324 patients who were not likely to have LS tumours according to molecular test results, 243 did not fulfil the RBG (specificity 75%).

## Discussion

In this prospective study of unselected and consecutively diagnosed CRC patients, we assessed the performance of currently used clinical guidelines (AM II and RBG) against three separate molecular tumour tests (MSI, *BRAF* and *MLH1* methylation analyses) in the identification of possible LS. Half of the patients whose tumours were highly suspicious of LS did not fulfil the RBG (sensitivity 50%), and five of the eight patients who had possible LS according to AM II were not LS, on the basis of the results of molecular testing.

The low sensitivity of AM II could be expected from previous research (Vasen *et al*, 2007; Julie *et al*, 2008). The moderate PPV (38%) in detecting possible LS-associated tumours in this study may question the usefulness of the AM II criteria in identifying LS patients. Other studies have reported that molecular screening analyses (MSI and IHC) may identify CRC patients as being associated with LS, despite the failure of the AM II criteria to identify these patients (Hampel *et al*, 2005). Furthermore, studies have shown that the proportion of MMR gene mutations may range from 49 to 80% among families who fulfil the AM II criteria (Wijnen *et al*, 1997; Wagner *et al*, 2003).

The RBG were established to capture a high proportion of LS patients by selecting tumours that should be tested for the presence of MSI and/or IHC. Although not diagnostic, MSI is strongly associated with LS. In that perspective, the RBG sensitivity of 50% is disappointingly low. It is noteworthy that our estimate is also low compared with other population-based studies that have reported a sensitivity of ∼90% for RBG in identifying MSI-H tumours suspicious of being LS associated (Pinol *et al*, 2005; Vasen *et al*, 2007). Moreover, our results showed that only 7% of patients who were selected for MSI/ICH testing by the RBG were confirmed as being highly suspicious of LS by molecular tumour testing. Our results suggest that the RBG may not be as useful as expected in identifying patients who are eligible for further molecular tumour analyses (MSI and/or ICH).

It could be argued that the low sensitivity of the RBG and modest predictive values of the AM II suggest that the molecular tests of this study may not represent a good diagnostic standard for the detection of LS. However, it is well documented that ∼95% of CRC tumours associated with LS are MSI-H (Boland, 2006). Although a small subgroup with *MSH6* mutation may be MSS (Ku *et al*, 1999), *MSH6* mutations only account for <10% of MMR mutation defects, whereas *MLH1* and *MSH2* mutations account for more than 90% of MMR mutations in LS (Domingo *et al*, 2004). As 10–20% of sporadic CRC tumours are MSI-H, isolated MSI-H status cannot reliably confirm LS. However, methylation of MMR genes (mainly *MLH1*) strongly suggests that MSI-H tumours are sporadic (Niv, 2007). Two studies have reported possible germline methylations of *MLH1* and *MSH2* (Chan *et al*, 2006; Hitchins *et al*, 2007), but in one study, methylation could not be detected in other family members, and was therefore not likely to be caused by germline inheritance (Hitchins *et al*, 2007). Others have not been able to verify germline MMR methylation (Boland *et al*, 2008). Therefore, *MLH1* methylation is presumed to reflect an epigenetic mechanism that inactivates the *MLH1* gene (and possibly the *MSH2* gene), causing MSI-H in tumours not associated with LS. If *BRAF V600E* mutations are present in MSI-H tumours, the possibility of LS can be nearly excluded, but only approximately 40–50% of MSI-H sporadic CRC tumours are reported to have *BRAF* mutations (Wang *et al*, 2003). We therefore used a combination of these three molecular tumour tests as an alternative to germline mutation analyses to detect possible LS-associated tumours in this study.

The molecular tumour characteristics of the patients in our study seem to be as expected in an unselected group of CRC patients, as the distribution of various molecular and genetic variants is fairly similar to that observed in other population-based studies (de la Chapelle, 2004; Hampel *et al*, 2005). *BRAF* mutations are reported to be present in ∼15% of sporadic CRCs, which compares with our result of 17.3%. It has been reported that a large majority (43–87%) of MSI-H sporadic tumours have *BRAF* mutations, whereas only a small proportion (4–9%) of MSS tumours have *BRAF* mutations (Domingo *et al*, 2004; Kambara *et al*, 2004). In our study, the respective proportions were 67.7% (MSI-H) and 5.6% (MSS). Approximately 10–20% of sporadic CRC tumours have been reported to be MSI-H (Imai and Yamamoto, 2008), and the corresponding proportion in our study was 17%. The prevalence of CRCs associated with LS has been 3–5% in most studies (de la Chapelle, 2004; Lynch *et al*, 2004; Vasen *et al*, 2007), and on the basis of molecular tumour tests, we estimated a prevalence of 3.6%. Most studies of unselected CRC patients have analysed *MLH1* and *MSH2* genes, as these are most commonly mutated in LS (de la Chapelle, 2004; Domingo *et al*, 2004; Julie *et al*, 2008). Pinol *et al* (2005) performed only IHC and MSI analyses among unselected CRC patients to identify *MSH2* and *MLH1* germline mutation carriers, whereas we also included molecular analyses for the detection of mutations of *MSH6* and *PMS2*. Many studies have used highly selected groups of CRC patients, or included patients from hereditary cancer clinics (Young *et al*, 2001; Hitchins *et al*, 2007) Differences in study design will typically complicate comparisons with the results of population studies.

Molecular tumour analyses may improve the diagnostic accuracy of LS (Vasen *et al*, 2007; Julie *et al*, 2008), and several molecular analyses have been used (Young *et al*, 2001; Deng *et al*, 2004; Kambara *et al*, 2004; Koinuma *et al*, 2004; Pinol *et al*, 2005; Imai and Yamamoto, 2008). Immunohistochemistry has the advantage of identifying the underlying MMR gene defect, and germline analyses can be targeted to that specific MMR gene. However, IHC can only identify loss of proteins due to known MMR gene mutations, whereas MSI analyses can indicate other potentially pathogenic MMR genes (Hampel *et al*, 2005; Hendriks *et al*, 2006; Lynch *et al*, 2007; Zhang, 2008).

Ideally, the combination of the three molecular tumour tests that we used should be validated against germline analyses. It is a weakness of this study that the results of germline analyses were not available. On the other hand, there are also weaknesses related to the performance and interpretation of germline analyses (de la Chapelle, 2004; Lynch *et al*, 2004), and the existence of other, not yet detected, genetic causes of LS cannot be excluded.

The six microsatellite markers that we used have been validated against MSI analyses, and have been found to perform well (Boland *et al*, 1998; Young *et al*, 2001; Hampel *et al*, 2005). Some studies have restricted the analysis to one marker (Pinol *et al*, 2005), and others have used some of the markers that we used. The panel of markers recommended by Boland *et al* (1998) has been used in several studies.

A reliable family history is an important criterion, both for the RBG and AM II (de la Chapelle, 2004; Lynch *et al*, 2004; Strate and Syngal, 2005). Many studies have shown that the quality of family history is suboptimal. This may be caused by patients’ lack of knowledge about their family's medical history, small-size families and other factors, but also because the importance of family history in CRC is often neglected by the clinician (Sanchez *et al*, 2008; Tranø *et al*, 2009). It is a strength of this study that the investigators who obtained family history were blinded to the results of the molecular tumour analyses and to pathology reports. Nonetheless, family history could be misclassified and could thereby influence the results of our study. Thus, if patients systematically underreported family history, or if family history was underestimated by the investigators, the sensitivity of the RBG could be underestimated. Similarly, the PPV of the AM II could also be underestimated. However, this potential bias was of great concern, and every effort was made to record family history accurately.

Age at diagnosis of the patient or of affected family members is an essential criterion for LS (de la Chapelle, 2004; Lynch *et al*, 2004; Strate and Syngal, 2005). However, the importance of early age at diagnosis seems to be less emphasised now than previously (Lynch *et al*, 2004; Boland, 2006). Whether histopathological features associated with LS are age dependent is not clear (Young *et al*, 2001; Boland *et al*, 2008), and the age limit of 60 years at diagnosis in the third criterion of the RBG may therefore be questioned (Jenkins *et al*, 2007).

The identification of LS remains a challenge in the clinical work up of CRC patients, and there is no single diagnostic test available that can reliably identify patients with LS. In addition to using clinical criteria, histopathological features and molecular screening analyses could be incorporated in the assessment of all CRC patients. Sanchez *et al* (2008) reported that the highest detection rate of LS in CRC patients was obtained by the combined efforts of the pathologist and the clinician.

## Conclusions

In a series of unselected CRC patients, we assessed the performance of the RBG and the accuracy of the AM II against three molecular tumour tests that may fairly accurately detect LS. The test combined MSI, *BRAF* mutation and *MLH1* methylation analyses.

The results showed that compared with molecular test results, the clinical guidelines performed poorly in identifying possible LS-associated tumours (RBG) or LS patients (AMII). The AM II criteria identified a large proportion of tumours as LS that were not associated with LS according to the molecular test results, and the RBG failed to select 50% of patients whose tumours were likely to be associated with LS.

Assuming that the molecular test results are fairly accurate, these results suggest that screening of CRC tumours by these molecular tumour tests could be offered to all CRC patients to improve the diagnosis of LS.

## Figures and Tables

**Table 1:** Criteria of the Revised Bethesda Guidelines (One of the criteria is sufficient to select the patient/family for further molecular testing for Lynch syndrome)

1. CRC diagnosed in a patient <50 years of age
2. Presence of synchronous, metachronous colorectal or other Lynch-related tumours,^a^ regardless of age
3. CRC with MSI-H phenotype^b^ diagnosed in a patient aged <60 years
4. Patient with CRC and a first-degree relative with a Lynch syndrome-related tumour, with one of the cancers diagnosed at age <50 years
5. Patient with CRC with two or more first- or second-degree relatives with Lynch syndrome-related tumour, regardless of age

Abbreviations: CRC=colorectal cancer; MSI-H=microsatellite instability-high.

a Lynch syndrome-related tumours: colorectal, endometrial, stomach, ovarian, pancreas, ureter, renal pelvis, biliary tract and brain tumours, sebaceous gland adenomas and keratoacanthomas, and carcinoma of the small bowel.

b Lymfocyte-infiltrating tumours, low grade or undifferentiated, Crohn's-like lymphocyte infiltration, the presence of mucin or signet cells in the tumours, and ‘cribiform growth pattern’.

**Table 2:** The Amsterdam II criteria for Lynch syndrome (All criteria must be fulfilled)

At least three relatives with colorectal cancer (CRC) or Lynch syndrome-associated cancer: cancer of the endometrium, small bowel, ureters and renal pelvis
One relative should be a first-degree relative of the other two
At least two successive generations should be affected
At least one tumour should be diagnosed before the age of 50 years

FAP (familial adenomatous polyposis) should be excluded.

Tumours should be verified by histopathological examination.

**Table 3:** Study participants according to categories of the Revised Bethesda Guidelines (Classified according to the ‘dominant’ criterion; that is, one patient can fulfil more than one of the criteria)

**Revised Bethesda Guidelines 2004**	**Number of patients**	**Proportion (%)**
1. CRC diagnosed in a patient <50 years	20	23.0
2. Presence of metachronous, synchronous colorectal or other Lynch-related^a^ tumours, regardless of age	14	16.1
3. CRC with MSI-H phenotype^b^ diagnosed before 60 years of age	21	24.1
4. Patient with CRC and a first-degree relative with a LS-related tumour, with one cancer diagnosed before 50 years of age	12	13.8
5. Patient with CRC with two or more first- or second-degree relatives with a LS-related tumour, regardless of age	20	23.0
Total	336	100.0

Abbreviations: CRC=colorectal cancer; LS=Lynch syndrome; MSI-H=microsatellite instability-high.

a Lynch syndrome-related tumours: colorectal, endometrial, stomach, ovarian, pancreas, ureters, renal pelvis, biliary tract and brain tumours, sebaceous gland adenomas and keratoacanthomas, and carcinoma of the small bowel.

b Lymfocyte-infiltrating tumours, low grade or undifferentiated, Crohn's-like lymphocyte infiltration, the presence of mucin or signet cells in the tumours, and ‘cribriform growth pattern’.

**Table 4:** Patient demographics, family history and tumour characteristics of patients with MSI-H, no BRAF mutation and no MLH1 methylation colorectal cancers

**ID**	**Age (years)**	**Gender**	**Family history**	**Tumour localisation**	**Histopatholgogy of tumour**	**Synchrounous cancer**	**Metachronous cancer**	**RBG**
1	81	M	4 FDRs; 2 Bro:Abd(67,76) 13 SDRs (4 SDR no knowledge about disease)	Right colon (Transverse colon)	Medium differentiation Muc:+ Sign:+ Lymf: ? ,Cro:+	No	No	Negative
2	69	F	9 FDRs Fa:CR(70);Mo:CR(67) 18 SDRs (2 MaUn:Abd (60,60)	Left (Rectosigmoid tumour)	Medium differentiated Muc: ?, Sign: ?, L= ?,Cro: ?	No	No	Negative
3	64	M	8 FDRs (Fa:CR(76); Mo:Ma(70) 11 SDRs (MaUn; MaAu; and MaGrandMa: Ca(old)	Right (Coecum)	Medium differentiated. Muc: −,Sign: −,Lymf: −, Cro: −	No	No	Negative
4	72	F	13 FDRs(Si:Abd(75),Si:Pa(75),Bro:Abd(66) 16 SDRs(PatAu:Ma(72)	Rectal cancer	Medium differentiated Muc: +,Sign: −,Lymf: +,Cro:+	No	No	Negative
5	87	M	Single child. No children. No knowledge of Pat family 1 FDR (father unknown) 3 SDR (no ca)	Right (Transverse colon)	Medium differentiated Muc: -,Sign: −,Lymf: −,Cro: −	No	No	Negative
6	75	M	No children 4 FDRs (1 Bro P(85)) 18 SDRs (no cancer)	Left (Sigmoid cancer)	Low differentiated Muc: +,Sign: −,Lymf: +,Cro: ?	No	No	Negative
7	87	F	Single child. No knowledge of father/Pat family 5 FDRs (Da:EN(38) Mo;Br(72)) 9 SDRs (no ca)	Right (Ascending colon)	Low differentiated Muc: −,Sign: −,Lymf: ?,Cro: −	No	Metachronous EN(47)	Positive: 2.Met LS-related cancer 4.1 FDR with LS-ass ca <50
8	58	M	No children Small size family and limited knowledge 3 FDRs: (Fa:CR(46)) 6 SDRs (MatGrandMa:’abd’(75), PatAu: Br	Right (Coecum)	Low differentiated Muc: +,Sign: −,Lymf: -,Cro: ?	No	No	Positive: 3.Histopath <60 years 4.1 FDR with CRC<50
9	55	M	5 FDRs (2<50 years) 15 SDRs(Mat cousin: Br(45)and lymf.ca(46)	Right (Transverse colon)	Medium differentiated Muc: +,Sign: −,Lymf: ?,Cro: +	No	No	3. Histopath <60 years
10	72	M	11 FDRs>40 years (Si:CR(58), Da:CR(38) 12 SDRs (no cancer)	Right and left (Liver flexure, sigmoid)	Medium differentiated Muc: −,Sign: −,Lymf: -,Cro: +	Yes (sigmoid)	No	2. Synchronous CR 4. 1 FDR with CRC<50
11	48	M	5 FDRs>50 years Mo;CR(54) Si:EN(55) 16 SDRs (no ca)	Right (Ascending colon)	Medium differtiated Muc: +,Sign: +,Lymf: ?,Cro:+	No	No	1.<50 years
12	46	F	6 FDRs>20 years (Si:UC(51), Mo:UC(48), Fa;P(75) 5 SDRs (MatGrandMo:Ur (>50)	Right (Transverse colon)	Medium differentiated Muc: -,Sign: −,Lymf: −,Cro:−	No	No	1.<50 years 5:>2 FDR or SDR with LS-ass ca

Abbreviations: Abd=abdomen or abdominal; Au=aunt; Br=brain tumour; Bro=brother; Ca=cancer of unknown origin; CR=colorectal; Cro=Crohn's-like aggregation of lymphocytes in tumour; Da=daughter; EN=endometrium; F=female; Fa=father; FDR=first-degree relative (mother, father, siblings, children); GrandFa=grandfather; GrandMo=grandmother; LS-ass ca=Lynch syndrome-related cancer; Lu=lung cancer; Lymf=lymphocytic infiltration of tumour; M=male; Ma=breast cancer; Mat=maternal; Mo=mother; Muc=mucinous differentiation >50% of secreting tumour cells; P=prostate; Pa=pancreas; Pat=paternal; SDR=second-degree relative (grandparents, aunts, uncles, grandchildren); Si=sister; Sign=signet cells; So=son; UC=uterine cervix; Un=uncle; Ur=ureter or cancer of the ureter/renal pelvis.

Lynch syndrome-related tumours=colorectal, endometrium, cancer of the small bowel and ureter or renal pelvic cancer.

**Table 5:** Comparison between the molecular test results and fulfilment of clinical guidelines

	**MSI status**	**BRAF mutation**	**MLH1 meth**	***N*=336**	**RBG positive**	**RBG negative**	**AM II positive**	**AM II negative**
LS	MSI-H	Wt	Neg	12	6	6	3	9
Non-LS	MSI-H	Wt	Pos	6	1	5	0	6
MSI-H sporadic CRC likely	MSI-H	V600E	Pos	39	11	28	0	39
Non-LS	MSS	Wt	Neg	260	63	197	5	255
Non-LS (sporadic CRC likely)	MSS	V600E	Neg	19	6	12	0	19
Total				336	87	249	8	328

Abbreviations: AM II neg=Amsterdam II criteria not fulfilled; AM II pos=Amsterdam II criteria fulfilled; CRC=colorectal cancer; LS=Lynch syndrome; MLH1 meth neg=absence of MLH1 methylation; MLH1 meth pos=methylation of MLH1; MSI=microsatellite instability; MSI-H=microsatellite instable tumour; MSS=microsatellite stable tumour; non-LS=CRC not associated with Lynch syndrome; RBG pos=Revised Bethesda Guidelines fulfilled; RBG neg=Revised Bethesda Guidelines not fulfilled; V600E=presence of the BRAF oncogene; Wt=absence of the BRAF oncogene.

**Table 6:** Performance of the Revised Bethesda Guidelines (2004) and accuracy of the Amsterdam II criteria against the molecular tumour analyses

	**Molecular tumour analyses**		
	**Lynch syndrome likely**	**Lynch syndrome not likely**	
*Revised Bethesda Guidelines (RBG)*			
Positive	6	81	87
Negative	6	243	249
	12	324	336
			
*Amsterdam II criteria (AM II)*			
Positive	3	5	8
Negative	9	319	328
	12	324	336

Abbreviations: CI=confidence interval; PPV=positive predictive value.

RBG: Sensitivity 50% (95% CI, 21–79%); specificity 75% (95% CI, 70–80%), PPV 7% (95% CI, 3–14%).

AM II: Sensitivity 25% (95% CI, 6–57%), specificity 98% (95% CI, 96–99%), PPV 38% (95% CI, 9–76%).

**Table 7:** Clinical characteristics, molecular screening test results and family history of patients fulfilling the Amsterdam II criteria

**ID**	**Age at CRC (years)**	**Gender**	**Tumour localisation**	**MSI status**	**BRAF**	**Methylation status**	**Molecular analyses conclusion**	**Tumour in relative (age at diagnosis in years)**	**Germline test result**
1	48	M	Right colon	MSI-H	Wt	Neg	LS likely	Mo:CR(54); Si:EN(55)	PMS2
2	46	F	Right colon	MSI-H	Wt	Neg	LS likely	Mo:EN(48); MatGrandMo:Ur(56); Fa:Pro(70) Si:CER(51);	MSH2
3	74	F	Rectum	MSS	Wt	Neg	Likely non-LS	Da:EN(48);Si:EN(49); MatGrandFa:Abd(70)	
4	75	F	Right colon	MSS	Wt	Neg	Likely non-LS	Mo:CR(46); Bro:CR(78);Si:Cr(79)	
5	67	M	Right colon	MSS	Wt	Neg	Likely non-LS	Bro:CR(35);Fa:Cr(85); PatGrandPa:Cr(60); PatUn:Abd(∼70); PatUn:Abd (∼70); PatAu:Abd(∼70); PatAu:Abd(∼70)	
6	59	M	Rectum	MSS	Wt	Neg	Likely non-LS	Bro:CR(29); Mo:EN(40); MatAu:CR(58);MatAu:CR(63) Fa:Pro(65); 11 MatAu/MatUn:Unkn	
7	81	M	Right colon	MSS	Wt	Neg	Likely non-LS	Bro:CR(70); Pat Un:CR(53); Pat Un CR(27); Pat Un:CR(50); Fa: ‘Bile/gall bladder’ Mat Un;Lung(smoker)	—
8	72	M	Right and left colon (synch)	MSI-H	Wt	Neg	LS likely	Si: CR(58). Da: CR(38); Pat.Un CR(53); Pat.Un Abd(50)	Germline test MLH1, MSH2, MSH6, PMS2, negative

Abbreviations: Abd=abdomen; Au=aunt; Bro=brother; CR=colorectal; CRC=colorectal cancer; Da=daughter; EN=endometrium; Fa=father; GrandFa=grandfather; GrandMo=grandmother; Mat=maternal; methylation status=promotor methylation of MLH1; Mo=mother; MSI=microsatellite status; MSI-H=microsatellite high; MSS=microsatellite stable; neg=no methylation; Pat=paternal; pos=methylation found; Pro=prostate; Si=sister; So=son; Un=uncle; Unkn=unknown; Ur=ureter or cancer of the ureter/renal pelvis; UT CER=uterine cervix; wt=no BRAF mutation.

*Molecular screening analyses conclusion*: LS=if possible LS; non-LS=if likely sporadic CRC.

Lynch syndrome-associated tumours=colorectal, endometrium, cancer of the small bowel and ureter or renal pelvic cancer.

## Appendix A

Laboratory methods:

Micro-dissection of paraffin-embedded tumour tissue and normal mucosa and analyses for microsatellite instability (MSI) were performed on matched pairs of tumour DNA and DNA from normal mucosa or lymphocyte samples. DNA was extracted using BioRobot EZ1 workstation from Qiagen (Hilden, Germany), according to the manufacturer's protocol (http://www.qiagen.com).

Multiplex MSI analysis was performed comparing normal *vs* tumour tissue at six microsatellite loci (TGF-*β*-IIR, BAT25, BAT40, D5S107, D5S406 and D13S153) (Boland *et al*, 1998). If two or more of the markers (30%) were shifted in size in tumour tissue, they were classified as microsatellite unstable (MSI-High). (Aaltonen *et al*, 1998; Loukola *et al*, 2001).

The *BRAF* mutation analysis was performed by screening for a heteroduplex in exon 15. Subsequently DNA sequencing was carried out to verify the p.Val600Glu mutation. Heteroduplex analysis was performed by high-resolution melting (HRM) analysis on the Rotor-Gene 6000 (Corbett Research, Mortlake, NSW, Australia) machine. BRAF exon 15 primers were similar for HRM and sequencing; forward primer TCATAATGCTTGCTCTGATAGGA and reverse primer GGCCAAAAATTTAATCAGTGGA. In HRM analyses, a 10 *μ*l PCR reaction was performed using master mix Sensimix HRM Kit (Quantace, London, UK), with an annealing temperature of 53°C. Three parallels were analysed in the HRM analyses with a melting temperature between 70 and 85°C.

Methylation analyses were performed using the SALSA MS-MLPA kit ME011 mismatch-repair genes according to the manufacturer's protocol (MRC-Holland, Amsterdam, The Netherlands) http://www.mrc-holland.com).
